# Utilization of growth monitoring and promotion services and associated factors among children aged 0-23 months in Banja District, Northwest Ethiopia 2020: A cross-sectional study

**DOI:** 10.1371/journal.pone.0259968

**Published:** 2021-11-15

**Authors:** Alex Yeshaneh, Temesgen Fentahun, Tefera Belachew, Anissa Mohammed, Daniel Adane

**Affiliations:** 1 Departments of Midwifery, College of Medicine and Health Sciences, Wolkite University, Wolkite, Ethiopia; 2 School of Public Health, College of Medicine and health science, Wollo University, Dessie, Ethiopia; 3 Nutrition and Dietetics Department, Faculty of Public Health, Institute of Health, Jimma University, Jimma, Ethiopia; University of the Punjab, PAKISTAN

## Abstract

**Background:**

Growth monitoring and promotion are the basic malnutrition preventive strategies usually used to assess the growth of children using anthropometric measurements in comparison with world health organization standards. However, the utilization of growth monitoring and promotion services is inadequate in most developing countries. Therefore, this study aimed to assess the utilization of growth monitoring and promotion service and associated factors among children aged 0-23-month in Banja District, Northwest Ethiopia, 2020.

**Methods:**

A community-based cross-sectional study was conducted from February 2 to April 1, 2020. A total of 572 children were selected using a simple random sampling technique. Data were collected using structured and pre-tested interviewer-administered questionnaires. Data were entered into Epi data version 4.6 and analyzed using the statistical package for social science (SPSS) version 25. Both binary and multivariable logistic regression analyses with a 95% confidence level were used to identify the associated factors. Statistical significance was set at p <0.05.

**Results:**

This finding revealed that the proportion of growth monitoring and promotion services utilization was 38.9% [95%CI: 34.8%, 43.0%]. Child age from 0-11 months [AOR = 4.98 (95% CI: 2.75,8.37)], mothers who can read and write Amharic language [AOR = 2.04 (95%CI: 1.02,4.08)], know the benefits of weighing their child monthly [AOR = 2.9 (95%CI: 1.23, 6.94)], presence of growth monitoring service nearby [AOR = 3.2 (95%CI: 1.59,6.31)] and monthly income ≥2000 Ethiopian birr [AOR = **1.75**(95% CI = 1.08, 3.02)] were some of the factors significantly associated with utilization of growth monitoring and promotion services.

**Conclusion and recommendation:**

The findings indicate that utilization of growth monitoring and promotion services is mainly affected by child age, mother/caregiver ability to read and write Amharic language, having maternal information on the benefit of the weighing child, presence of service nearby health facility, and mother/caregiver monthly income. Preparation of growth monitoring charts in local language (Awigna) and creating awareness on the proper utilization of growth monitoring and promotion services is strongly recommended.

## Introduction

Child growth is an insightful display of childhood health, and is used to assess nutritional and health status [1]. Weight is a sensitive indicator used to assess growth [2, 3]. Growth monitoring is a monthly evaluation of the child’s growth in comparison to the world health organization standard using anthropometric measurements to identify growth faltering before the child reaches the status of malnutrition [4, 5].

Growth monitoring and promotion (GMP) is a prevention strategy that monitors measures, interprets and analyzes the possible reasons for adequate or inadequate child growth. It also facilitates communication and interaction, generates adequate action, improves the nutritional status of the child and reduces mortality and morbidity in children [4, 6].

In most countries of the world, the attendance rate, promotion and educational effectiveness towards GMP are very low and the growth chart is poorly understood by mothers. Few studies have addressed the relationship between the implementation of GMP programs and subsequent changes in caring practices [1, 7]. Effective growth-monitoring activities are not easily implemented and local realities are not often considered when making decisions about the inclusion of growth monitoring in national programs [1]. According to different pieces of evidence of poor growth monitoring, mothers’ illiteracy, poor parental intention for GMP, misunderstanding of the chart by health workers and poor intention of mothers for the service users were some of the shortlisted factors for GMP utilization [1, 8, 9].

Many countries have implemented GMP programs, resulting in a decrease in malnutrition. To improve child nutritional status, the Ethiopian government has been applying monthly GMP services at a community level using health extension programs through the implementation of the Sequota declaration and health sector transformation plan which aimed to stress nutritional counseling, early disease detection and treatment [2, 9–13].

Although many efforts have been made to reduce child mortality secondary to poor GMP utilization in Ethiopia, GMP utilization in health facilities is low [9, 10]. There is also a limited study conducted in Ethiopia in the Amhara region on the utilization of GMP services and associated factors among children aged 0-23 months [6]. Therefore, this study aimed to assess the utilization of GMP services and associated factors among children of 0-23 months in Banja District, Northwest Ethiopia.

## Method and materials

### Study design and setting

A community-based cross-sectional design was conducted from February 2 to April 1, 2020, in the Banja district of Northwest Ethiopia. Banja district is located 452 km from Addis Ababa (capital city of Ethiopia) and 199 km from Bahr Dar (capital city of Amhara regional state). Injibara is the administrative center of the district. The district is bordered Southwest by Ankesha district, West by Guangua, North by Fagita and on the East by Guagusa district and West Gojjam. The district had a total of 27 Kebele, 1 general hospital, 5 health centers, 27 health posts, 27 health officers, 44 nurses, 7 health information technicians and 636 health developmental armies. It also has 25 rural and 2 urban administrative kebeles with a total population of 99962 of which, 5048 were children less than two years of age [14].

### Populations

The source populations were **a**ll mother-child pairs of 0–23 months in Banja districts whereas all mother-child pairs with 0–23 months at selected Kebele during the study period were the study populations.

### Sample size determination

The minimum sample size was determined using a double population proportion formula and it was calculated using Epi Info™ version 7 by considering the assumptions (80% power of the study, 95% confidence level, 1:1 ratio) and proportion of outcome for the variable occupation (41.1% exposed and 53% non-exposed). After adding a 10% none response rate the final sample size for the study was **572** [15] (Table 1).

**Table 1:** Sample size determination to assess GMP utilization, 2020.

S. no	Factors	Assumptions						Sample size	Ref
		Ratio	Power	CI	OR	Proportion of outcome			
						Non-exposed	Exposed		
1	Place of delivery	1:1	80%	95%	3.01	10%	33%	224	[6]
2	Occupation	1:1	80%	95%	1.68	53%	41.1%	520	[15]
3	Education	1:1	80%	95%	1.99	52.4%	35.5%	306	[15]

### Dependent variable

❖ GMP utilization

### Independent variables

❖ Socio-demographic factors: Age of mothers, sex of the child

Head of household, ethnicity, religion, educational level, marital status, family size

❖ Maternal knowledge of GMP and IYCF…

### Sampling technique and procedures

All 27 administrative Kebeles from the Banja district were included to obtain the desired sample size. Initially, eight Kebeles (30%) were selected using the lottery method. A sampling frame containing a list of 1736 mother-child pairs less than 2 years old along with their date of birth and house number was obtained from the community health information system and respective Kebele. Then all listed households were coded by numeric code and 572 households were picked by lottery method. If we got two children who were less than 2 years old (0-23 months) within the household, a child with a smaller age should be taken. If we get twins within the households, pick either of the children using the lottery method. After population size to proportional allocation was performed, the sampled populations were selected using a simple random sampling technique.

### Operational definitions

**The regular weighing of children:** was measured when a child has a history of GM services utilization in respect to age such as, at least once for 0 months, two times for 1-3 months, five times for 4-11 months, and four times per year for 12-23 months and the finding should be plotted /recorded on the child GMC [6].

**Utilization of GMP services:** was measured when the respondents fulfilled the key indicators such as availability of the growth monitoring card, presence of regular weighing, getting nutritional advice from HCP, read GMC graph, and knowing the benefits of regular weighing at the time of data collection whereas those who did not fulfil these key indicators were considered as not properly utilizing GMP [6].

### Data collection tools

A pretested, semi-structured interviewer-administered questionnaire was prepared by reviewing relevant works of literature. The tool consisted of socio-demographic characteristics, knowledge-related, health service-related and media-related components. In the data collection process, four experienced clinical nurses and two public health officers were recruited and trained for data collection and supervision respectively.

### Data quality control

To ensure quality, the questionnaire was translated into the local language(*Amharic* and *Awigna*) by experts. Finally, before data collection, it was re-translated back to English to verify consistency. Before starting the actual data collection, one day of extensive training was given to the data collectors and supervisors. A pre-test for appropriateness and feasibility of the tool was conducted on 5% of the total sample size at Ankesha district, Bekefta Kebele and all necessary modifications and amendments were done accordingly. The reliability test or Cronbach’s alpha correlation coefficient of greater than or equal to 0.7 was used to check the inter-item consistency of the tool. The data collection team was communicated and discussed with principal investigators if they face any challenges during the data collection period daily. After data collection or before analysis, all collected data were checked for completeness.

### Data processing and analysis

Data were coded, cleaned, edited and entered into Epi-data version 4.6 and exported to SPSS version 25.0 for statistical analysis. The presence of an association between explanatory and outcome variables was ascertained using binary logistic regression analysis. The goodness of fit was tested by the log-likelihood ratio (LR). To control all possible confounders all variables with P<0.2 in the binary analysis were included in the final model of multivariable analysis. Variables with a standard error of > 2 were dropped from the multivariable analysis. To see the correlation between independent variables, the multi-collinearity test was carried out by using collinearity statistics. In a multivariable logistic regression model adjusted odds ratio determined with a 95% confidence level was used to assess the strength of association and those variables with a P-value < 0.05 were deemed to declare statistical significance. Then, the finding was presented by using simple frequencies, summary measures, tables, figures and texts.

### Ethical clearance and consent to participate

Ethical clearance for the study was obtained from the ethical review committee of Wollo University College of Medicine and Health Sciences, School of Public Health. An official letter was sent to the Awi zone health department and Banja district health office. The data collection was begun after obtaining a consent and cooperation letter from the Banja district health office. The study purpose, procedure and duration, rights of the respondents and data safety issues, possible risks and benefits of the study were clearly explained to each participant using the local language. Then before the commencement of the study, all participants gave their informed written consent. Participation in this study was purely voluntary and there was no monetary gain. The mothers/caregivers were expected to be free to withdraw from the study without any penalty. No compensation was offered for participation in the study. All the participants’ response was kept confidential by using the information only for the study and storing the study in a closed file.

## Results

### Socio-demographic characteristics of respondents

In this study, a total of 561 respondents have participated with a response rate of 98%. The majority of 460 (82%) of the study participants were rural residents. Nearly half (50.8%) of the study participants were female children and almost all (96.6%) of children were delivered at a health facility. Two hundred forty-four (43.5%) of children were found in the age group of 12-24 months. Three hundred thirty-three (59.4%) of mothers and 72.9% of the husbands have been working in farming. fife hundred forty-six (97.3%) were orthodox Christian religious followers. More than half (54.1%) of mothers and three-fourth (74.5%) of the husbands had attended the primary level of education (Table 2).

**Table 2:** Socio-demographic characteristics of the study participants in Banja District, Northwest Ethiopia, 2020 (n = 561).

Variables	Category	Frequency	Percent
Child age in months	0-5	173	30.8
	6-8	68	12.1
	9-11	76	13.6
	12-24	244	43.5
Sex of the child	Male	276	49.2
	Female	285	50.8
Age of the mothers	15-19 years	16	2.9
	20-24 years	58	10.3
	25-29 years	172	30.7
	30-34 years	180	32.1
	35-39 years	93	16.6
	>39 years	42	7.5
Religion	Orthodox Christian	546	97.3
	Protestant	15	2.7
Residency	Rural	460	82.0
	Urban	101	18.0
Ethnicity	Agaw	346	61.7
	Amhara	215	38.3
Family size	<4	32	5.7
	4-7	480	85.6
	>7	49	8.7
Number of children <2 years within the household	One	560	99.8
	Two	1	0.2
Number of birth	1	75	13.4
	2-3	229	40.8
	4-5	205	36.5
	>5	52	9.3
Place of birth	Home	19	3.4
	Health facility	542	96.6
Head of the household	Husband	531	94.7
	Wife	30	5.3
Relation (mother type) with a child	Biological	542	96.6
	Caregiver/legal	19	3.4
Educational status of the mother	No formal education	263	46.9
	Primary	190	33.9
	Secondary	78	13.9
	College and above	30	5.3
Occupation of the mother	Housewife	103	18.4
	Farmer	333	59.4
	Merchant	69	12.3
	Government employee	17	3.0
	Private employee	20	3.6
	Daily workers	19	3.4
Marital status	Single	21	3.7
	Married	510	90.9
	Divorced	13	2.3
	Widowed	17	3.0
Husband’s Educational level (n = 510)	No formal education	130	25.5
	Read and write	40	7.8
	Primary	158	31.0
	Secondary	116	22.8
	College and above	66	12.9
Husband’s Occupation	Farmer	371	72.9
	Merchant	67	13.1
	Government employee	45	8.8
	Private employee	14	2.7
	Daily workers	13	2.5
Family monthly income	<2000 Ethiopian Birr(ETB)	307	54.7
	2000-5000 ETB	215	38.3
	>5000 ETB	39	7.0

### Mothers or caregivers knowledge towards IYCF

About 44.6% of respondents got nutritional advice from health care providers. Nine out of ten (87.8%) respondents knew that breastfeeding should start within one hour after birth and nearly three-fourth (74.2%) of respondents knew the recommended age/time of exclusive breastfeeding. Nearly all (99.1%) respondents knew the time when breastfeeding should be terminated. Nearly three-fourths (74.2%) of respondents knew the standard period when complementary feeding has to be started (Table 3).

**Table 3:** Mothers/caregivers knowledge on IYCF in Banja District, Northwest Ethiopia, 2020 (n = 561).

Variables	Category	Frequency	Percentage
Nutritional advice from the health care providers	Yes	250	44.6
	No	311	55.4
The first thing to be given after birth	Breast milk	561	100
Time of breastfeeding initiation	Within **1** hour	492	87.7
	> **1** hours	69	12.3
Duration of the exclusive breastfeeding	< 6 months	47	8.4
	6-8 months	416	74.2
	> 8 months	98	17.5
Continuation of breastfeeding	0- 12 months	47	8.4
	12-24 months	5	0.9
	> 24 months	556	99.1
Time of complementary feeding initiation	< 6 months	47	8.4
	6-8 months	416	74.2
	> 8 months	98	17.5
Frequency of exclusive breastfeeding/day	<8 times	99	17.6
	≥8 times	462	82.4
Daily frequency of complementary feeding for a 6-8-month-old child	Once	2	0.4
	2-3 times	559	99.6
Daily frequency of complementary feeding for a 9-23 month child	<3 times	2	0.4
	3-4 times	559	99.6
Daily frequency of complementary feeding for non-breastfeeding 6-23 months child	<3 times	2	0.4
	3-4 times	559	99.6
Breast milk/food for a sick child	Increased than usual	392	69.9
	The same as usual	169	30.1

### Mothers/caregivers knowledge towards growth monitoring

Of the total respondents, 95.5% knew about family health cards and only 4.3% of them mention the importance of growth monitoring cards as growth monitoring follow-up. Only 38.9% of children get weighing services regularly based on their age. More than three-fourth (81.1%) of respondents couldn’t read the growth monitoring chart. Among this, the majority (35.5%) of them have mentioned the reason as growth monitoring card is written in Amharic and English language **(Table 4).**

**Table 4:** Knowledge of growth monitoring among mothers/caregivers of 0-23-month-old children in Banja District, Northwest Ethiopia, 2020 (n = 561).

Characteristics	Category	Frequency	Percentage
Know a family health card	Yes	536	95.5
	No	25	4.5
Importance of a family health card (n = 536)	GM & vaccination follow-up	251	46.8
	Growth monitoring follow-up	23	4.3
	Vaccination follow-up	262	48.9
Know benefit of monthly weighing a child	Yes	461	82.2
	No	100	17.8
What is the importance of monthly weighing a child (n = 461)	Growth follow-up	301	65.3
	Health follow-up	160	35.7
Presence of a growth monitoring card	Yes	561	100
Immunization status of vaccines based on the age of a child	Appropriate for age	553	98.6
	Not appropriate for the age	8	1.4
Weight of a child based on their age	Appropriate for age	218	38.9
	Not Appropriate for age	343	61.1
Reasons for not weighing a child	Lack of health care provider	96	28.1
	Weighing service is delivered with EPI	167	48.8
	Home to the facility is distant	21	6.1
	No time to go/busy	7	2.0
	It is not important	45	13.2
	I do not know	6	1.8
Able to read a growth monitoring card	Yes	106	18.9
	No	455	81.1
Who thought you about the reading of the graph (n = 106)	Health care provider	59	55.7
	Myself	40	37.7
	From other mothers	1	0.9
	Family member, from media	6	5.7
If the graph becomes decreased, it indicates (n = 106),	Not appropriate weight for age	105	99.1
	Appropriate weight for age	1	0.9
If the graph becomes similar, it indicates (n = 106)	Increasing weight for age	1	0.9
	Appropriate growth	61	57.4
	I do not know	44	41.5
If the graph becomes increases, it indicates (n = 106)	Child weight for age is increasing	105	99.1
	I do not know	1	0.9
Reason for not able to read a growth monitoring card (n = 455)	Growth monitoring card was written by Amharic and English	159	34.9
	Education was not given	153	33.6
	I am not worried about it	12	2.6
	Knowledge gap	5	1.1
	I do not Know	126	27.7

### Utilization of growth monitoring and promotion

The key indicators used to assess utilization of GMP in this study includes the availability of the family health card (whether it had been plotted or not), regular weighing, getting nutritional advice from a health care provider or knowledge on IYCF and GMP and able to understand growth monitoring graph or know the benefits of regular weighing. Based on this, the respondent who fulfills the key indicators were labeled as proper utilization of GMP and those who did not fulfill these were not properly utilizing GMP. Therefore, the finding of this study showed that 38.9% [95% CI = 34.8, 43] of the study participants have properly utilized the GMP service **(Table 5).**

**Table 5:** Utilization of growth monitoring and promotion service among 0-23-month-old children in Banja District, Northwest Ethiopia, 2020 (n = 561).

Characteristics	Level of the utilization of GMP service	
	Yes (%)	No (%)
Availability of the growth monitoring card	561(100%)	-
Utilization with regular weighing	213(38.0%)	348(62.0%)
Getting nutritional advice from health care providers	250 (44.6%)	3111(55.4%)
Can you read the growth monitoring graph/chart	106(18.9%)	455(81.1%)
**Proper utilization of GMP service**	218 (38.9%)	343(61.1%)

### The characteristic feature of accessible health services

All respondents reported that they have health facilities nearby their homes. Only 7.5% and 2% of respondents went to the health facility for weighing a child and for getting nutritional advice respectively. More than four-fifth (87.9%) of respondents got a growth monitoring service nearby a health facility **(**Table 6**).**

**Table 6:** Accessibility of health facility for mothers/ caregivers in Banja District, Northwest Ethiopia, 2020 (n = 561).

Variables	Category	Frequency	Percentage
Time is taken to reach the nearest health facility	< 2 hour	526	93.8
	≥2 hours	35	6.2
Ever went to the health facility for weighing a child	Yes	42	7.5
	No	519	92.5
Ever went to the nearest health facility to get nutritional advice	Yes	11	2.0
	No	550	98.0
Ever went to the nearest health facility for a vaccine	Yes	561	100
Ever went to the nearest facility to get care for a sick child	Yes	561	100
Ever gone to the nearest health facility to get the deworming drugs	Yes	111	19.8
	No	450	80.2
Have you ever get health service in the public health facility	Yes	561	100
Have you ever get health service in the private health facility	Yes	61	10.9
	No	500	89.1
Availability of growth monitoring service in the nearest health facility	Yes	493	87.9
	No	68	12.1
Is weighing service available in the nearest health facility	Yes	522	93.0
	No	39	7.0
Is nutritional advice/IYCF service available in the nearest health facility	Yes	487	86.8
	No	74	13.2
Is vaccination service available in the nearest health facility	Yes	561	100

### Factors affecting growth monitoring and promotion service utilization

In multivariable logistic regression, all significant variables in binary logistic regression were adjusted. The result showed that the age of children in months, the ability of mother/ caregiver to read and write the Amharic language, having information on the benefit of a monthly weighing of the child, mother’s knowledge on the importance of family health card, availability of GMP service and monthly income were significantly associated with utilization of GMP service.

In this study, children less than 12 months were five times more likely to utilize GMP service as compared to children 12-24 age groups [AOR = 4.98(95% CI: 2.75-8.37)]. Those mothers who can read and write Amharic language had 2.04 times more likely to utilize GMP service as compared to their counterparts[AOR = 2.04(95% CI: 1.02, 4.08)]. This study found, those respondents who knew the benefits of monthly weighing their child were 2.9 times more likely to utilize GMP service than who do not**[**AOR = 2.9(95% CI: 1.23,6.94)]. Those who received GMP service nearby health facility were 3.2 times more likely utilize than their counterparts [AOR = 3.2(95% CI: 1.59, 6.31)]. The monthly income was also another significant factor which contributed for utilization of GMP service. Respondents who had monthly income ≥2000 Ethiopian birr were 1.75 times more likely utilize the GMP service [AOR = **1.75**(95% CI = 1.08, 3.02)] (Table 7).

**Table 7:** Binary and multivariable logistic regression analysis for factors of GMP services utilization among children 0–23 months in Banja District, Northwest Ethiopia, 2020 (n = 561).

Variables	Category	GMP Utilization		OR with 95% CI	
		Yes	No	COR	AOR
Age of child in months	0-11	192	125	12.9(8.1-20.5)	4.98 (2.75,8.37)**
	12-24	26	218	1	1
Able to read & write the Amharic language	Yes	79	42	4.07(2.66-6.23)	2.04 (1.02,4.08)*
	No	139	301	1	1
Accessibility of media	Yes	131	141	2.16(1.53-3.05)	1.23 [0.79,1.92]
	No	87	202	1	1
Know the importance of a family health card	Yes	141	110	3.65(2.54-5.26)	1.83 (0.17,20.33)
	No	74	211	1	1
Know the benefit of weighing monthly child	Yes	203	258	4.46(2.5-8.0)	2.9 [1.23, 6.94]*
	No	15	85	1	1
Availability of growth monitoring Service nearby home	Yes	209	284	4.82(2.34-9.95)	3.2 [1.59,6.31]*
	No	9	59	1	1
Presence of IYCF service	Yes	207	280	4.23(2.18-8.23)	2.01(0.72,5.60)
	No	11	63	1	1
Educational status	Uneducated	203	60	1	1
	Educated	140	158	3.82(2.65-5.5)	1.77(0.72,4.37)
Monthly family income	<2000	194	328	1	1
	≥2000	24	15	2.705(1.4-5.3)	1.75 (1.08,3.02)*
Number of birth	1-3	146	158	2.37(1.67-3.4)	0.87 [0.38,1.56]
	>3	72	185	1	1

Note

*significant at p- value< 0.05

**significant at p-value < 0.001, AOR: Adjusted odds ratio, CI: Confidence interval, COR: Crude odds ratio, and 1 = reference.

## Discussion

This study revealed that utilization of growth monitoring and promotion service was found to be 38.9% with 95% CI [34.8-43.0]. This finding is lower than a study conducted in Accra (64%), Nyamir Kenya (53%), and Lawar (60%) [15–17]. Similarly, this study was lower than studies done in Butajira, South Ethiopia (44%), Areka South Ethiopia (56%) and Gondar Northern Ethiopia (50%) [3, 18, 19]. The difference might be due to the difference in study design, socio-demographic characteristics of respondents and study population. Specifically, GMP in Kenya was implemented for under five populations whereas GMP in Ethiopia was implemented among 0-23-month-old children. The majority of previous studies were also institution-based and the study subjects were those who came for vaccination service and sick children. This leads to a higher chance for utilization of growth monitoring and promotion services.

The utilization of growth monitoring and promotion services can be affected by various factors. Age of child, able to read and write the Amharic language, know the benefit of weighing child, availability of GMP service and monthly income were significantly associated with utilization of GMP service. Mothers with children aged less than 12 months were more likely to utilize GMP services compared to those with children aged 12–23 months. This finding was supported with the study done in Kenya [16], Ghana [20, 21] who found that there was a negative correlation between the age of the child and attendance to child health clinics. This might be due to GMP service is delivered along with an integrated vaccination in most of African countries [20] which allows most mothers to continue utilization of growth monitoring concurrently with immunization service. When most immunization service turnover after first birth day, attendance to child clinic decreases. However, this study contradicted with study done in southern part of Ethiopia in which the age group of 12-23 months found that more likely to utilize the GMP services as compared to infants [6].

Mothers who can read and write the Amharic language were two times more likely to utilize GMP than their counterparts. None of the studies support this finding. This is due to other studies didn’t use language parameters as a factor to determine GMP utilization. Findings of this study revealed that nearly 35% of caregivers could not read the growth monitoring chart because of growth monitoring chart was written in Amharic and English language and the dominant language in the study area is Awigna for 62% of mothers/caregivers. Language variation makes them not to understand the information displayed on the chart in which intern makes them not utilize GMP.

This study also found that the mothers who know the benefits of monthly child weighing were more likely to utilize the GMP service. This finding is comparable with study done in North Gondar [3]. However, study conducted in Ghana found that mothers’ knowledge regarding GMP was reversely associated with the utilization of GMP services in that those who had high level of knowledge were less likely to utilize GMP services [20]. In this finding more than half (55%) of mothers or care givers didn’t utilize the GMP service, even though they know the benefit of weighing child monthly. This implies that acquisition of knowledge may not necessarily enough to practice. Thus, interventions should not only concentrate on educating the mothers but they should also support them to overcome barriers that may be preventing them from utilizing GMP services.

Similarly, the availability of growth monitoring services nearby health facilities was also more likely utilize the GMP service. This finding is supported by the study conducted in Nyamir, Kenya [16], and Indonesia [22] where the availability of growth monitoring service nearby health facility was positively associated with utilization of GMP service. This is similarities might be due to those respondents who had nearby health centers might have better opportunities to access the services and information related to GMP.

Monthly income of mothers/ care-givers has also contribution for the utilization of growth monitoring and promotion service in this study. Respondents who had monthly income ≥2000 Ethiopian birr were more likely utilize the GMP service. This finding is in agreement with study done in southern Ethiopia where medium and high wealth index were more likely utilize GMP service [6]. This could be due to the financial constraint majority of mothers or care givers are engaged in daily works to serve the children rather than utilizing GMP service.

## Conclusion

The utilization of growth monitoring and promotion services in the study area was low. Factors like child age, mother/ caregiver ability to read and write Amharic language, the mother who knows the weighing benefit, availability of GMP service nearby health facility and monthly income were significantly associated with utilization of GMP service. To improve the utilization of growth monitoring and promotion services, a growth monitoring chart should be prepared in local languages (Awigna), health care providers should deliver sufficient information to the communities, the service delivery of growth monitoring and promotion should be provided separately from an expanded program of immunization.

### Limitations

This study shares the limitation of study design and our study was not a mixed type of study, it couldn’t address some important variables.

## Supporting information

S1 File This S1 file minimal data set of growth monitoring and promotion service. (SAV)

## Abbreviations

EDHS: Ethiopian Demographic Health Survey
EHSTP: Ethiopian Health Sector Transformation Plan
FHC: Family Health Card
GM: Growth Monitoring
GMP: Growth Monitoring and Promotion
HCP: Health Care Provider
HEW: health Extension Worker
HP: Health post
HSTP: Health Sector Transformation Plan
IYCF: Infant and Young Child Feeding
SDG: Sustainable Developmental Goal
SNNPS: Southern Nation and Nationality People State
SPSS: Statistical Package for Social Sciences
UNICEF: United Nations International Children Emergency Fund
UNDP: United Nations Development Program
WHO: World Health Organization
